# New Frontiers in the Treatment of Patients with HER2+ Cancer and Brain Metastases: Is Radiotherapy Always Useful?

**DOI:** 10.3390/cancers16132466

**Published:** 2024-07-05

**Authors:** Giuseppa Scandurra, Valentina Lombardo, Giuseppe Scibilia, Daniela Sambataro, Vittorio Gebbia, Paolo Scollo, Basilio Pecorino, Maria Rosaria Valerio

**Affiliations:** 1 Medical Oncology Unit, Cannizzaro Hospital, 95126 Catania, Italy; giuseppa.scandurra@unikore.it; 2 Department of the Medicine and Surgery, Kore University, 94100 Enna, Italydaniela.sambataro@unikore.it (D.S.); vittorio.gebbia@unikore.it (V.G.); paolo.scollo@unikore.it (P.S.); basilio.pecorino@unikore.it (B.P.); 3 Gynecology Unit, Giovanni Paolo II Hospital, 97100 Ragusa, Italy; 4 Medical Oncology Unit, Umberto I Hospital, 94100 Enna, Italy; 5 Medical Oncology Unit, CdC Torina, 90145 Palermo, Italy; 6 Gynecology and Obstetrics Unit, Cannizzaro Hospital, 95126 Catania, Italy; 7 Gynecology and Obstetrics Unit, Umberto I Hospital, 94100 Enna, Italy; 8 Medical Oncology Unit, Policlinico, University of Palermo, 90127 Palermo, Italy; mariarosaria.valerio@unipa.it

**Keywords:** breast cancer, brain metastases, radiotherapy, anti-HER drugs

## Abstract

**Simple Summary:**

Brain metastases are a major challenge for patients with HER2+ breast cancer. Traditional treatments, like radiotherapy, can help but often cause severe side effects and may not provide lasting control. Researchers are exploring new, more precise treatments, including antibodies, drug-antibody combinations, and small molecule drugs that can better penetrate the brain. These new therapies have shown promise in clinical trials, helping to control brain tumors more effectively and with fewer side effects than radiotherapy. The goal of this review is to improve treatments for HER2+ breast cancer patients who develop brain metastases, enhancing their survival rates and quality of life. The findings from this research could significantly impact the medical community by offering better alternatives to radiotherapy and improving how brain metastases are managed. This progress could provide new hope for patients facing this challenging condition and potentially transform treatment strategies in the future.

**Abstract:**

Brain metastases (BM) pose a significant challenge in the management of HER2+ breast cancer since almost 50% of patients with HER2+ breast cancer develop brain tumors. The complex process of brain metastases involves genetic mutations, adaptations and mechanisms to overcome the blood–brain barrier. While radiotherapy is still fundamental in local therapy, its use is associated with cognitive adverse effects and limited long-term control, necessitating the exploration of alternative treatments. Targeted therapies, including tyrosine kinase inhibitors, monoclonal antibodies, and antibody–drug conjugates, offer promising options for HER2+ breast cancer patients with BM. Clinical trials have demonstrated the efficacy of these agents in controlling tumor growth and improving patient outcomes, posing the question of whether radiotherapy is always the unique choice in treating this cancer. Ongoing research into novel anti-HER2 antibodies and innovative combination therapies holds promise for advancing treatment outcomes and enhancing patient care in this clinical scenario. This narrative review provides a comprehensive overview of traditional medical treatments, molecularly targeted therapy and investigational agents in the management of HER2+ breast cancer with BM, highlighting the evolving landscape and potential future directions in treatment strategies to improve patient survival and quality of life.

## 1. Introduction

**Figure 1:**
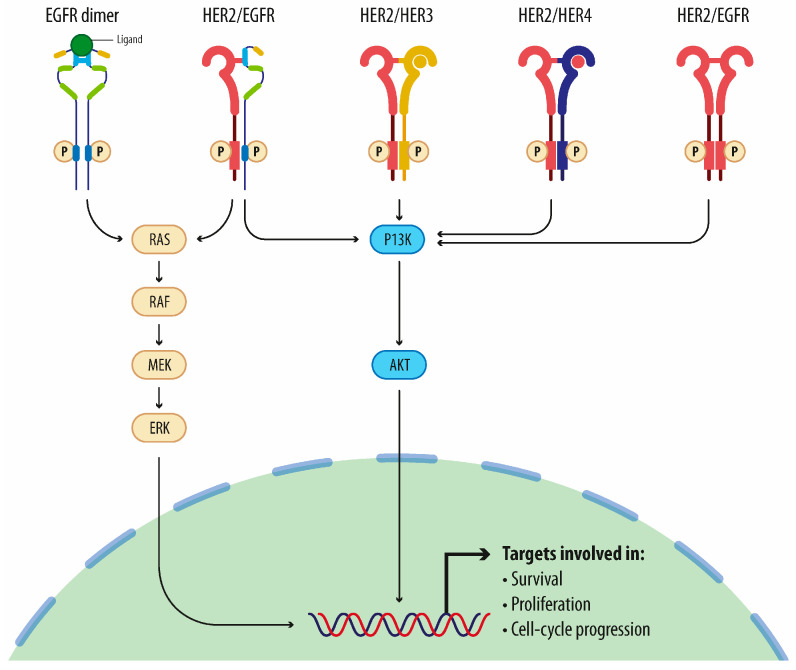
HER2 signaling pathway. Human epidermal growth factor receptors (EGFRs), including HER1 (EGFR), HER2, HER3 and HER4, are receptors that are characterized by extracellular ligand-binding domains, transmembrane domains and intracellular tyrosine kinase domains. Upon binding of specific ligands, the receptors undergo activation via phosphorylation. Notably, HER2 does not require ligand binding for activation. Dimerization triggers subsequent phosphorylation events, initiating downstream signaling cascades, such as the PI3K/AKT and RAF/MEK/MAPK pathways. Activation of these pathways is linked with cell survival, proliferation and progression through the cell cycle. This network of molecular interactions regulates critical cellular processes, contributing to cancer development [6]. Image created with BioRender.com.

Human epidermal growth factor receptor 2 (HER2) is overexpressed in 15–20% of breast cancers [1], leading to an increased number of HER2 dimers and hyperactivation of pathways like those of PI3K-Akt and MAPK, as shown in Figure 1 [2]. This leads to aggressive tumor growth and poorer outcomes compared to HER2-negative breast cancers [3]. In addition, its aggressivity is linked to the ability of these cancer cells to metastasize: approximately 50% of patients with human epidermal growth factor receptor 2-positive (HER2+) BC will develop brain metastases (BM) [4] and rates of BM across all metastatic BC (MBC) are increasing [5].

The process that leads to the migration of cancer cells from breast tissue to the central nervous system (CNS) is complex [7]. These cells must adapt to the environment of the brain and they have to overcome the blood–brain barrier (BBB).

Cancer cells undergo genetic mutations in order to metastasize; this process involves the selection of cancer cell subpopulations with traits that enhance their proliferation and survival in the microenvironment of the brain.

In addition, these cells became able to overcome the blood–brain barrier (BBB) to enter the brain parenchyma; this process involves mechanisms like secretion of enzymes that degrade the BBB components, interactions with endothelial cells and activation of signaling pathways [8].

Once entered, cancer cells must be able to escape immune surveillance mechanisms. Disruption of the BBB, facilitated by prior treatments like radiotherapy, may enhance cancer cell infiltration into the CNS [9].

Angiogenesis also disrupts the BBB, complicating treatment [10]. Future efforts aim to improve the quality of life and extend survival for patients with brain metastatic breast cancer (BMBC). In most cases, local therapy is chosen to treat brain metastases and current guidelines draw attention to surgery and radiotherapy (RT), which is considered extremely useful, especially for advanced stages of cancer. However, the biophysical impact of RT extends beyond tumor cells and it can result in toxic conditions for surrounding organs and tissues. 

For example, WBRT (whole-brain radiotherapy) can cause cognitive adverse effects, including somnolence, fatigue and memory and learning disabilities [11]. 

Moreover, the results of SRS (stereotactic radiosurgery) are not always satisfactory, especially in larger diameter BM. On one hand, a single high-dose radiotherapy may increase the risk of acute and late central nervous system toxicities. On the other hand, there are limitations of tolerated doses in peripheral critical organs [12]. 

Many studies suggest that the toxicity of RT plus targeted therapy is tolerable; actually, there are limited data on the effectiveness and toxicity of this combined therapy [13].

This narrative review was conducted by analyzing different aspects of HER2+ breast cancer, for example, brain metastases and specific treatment modalities such as targeted therapies, chemotherapy and radiotherapy.

We studied papers published between 1998 and 2023 in order to have a historical and recent look at research developments in this field. We considered a variety of study types to ensure a comprehensive understanding of the topic. This includes clinical trials, retrospective cohort studies, systematic reviews and meta-analyses. This approach allows an examination of both clinical evidence and research findings. 

The aim of the review is to focus attention on other ways to consider therapies and to take into consideration new treatments that are obtaining good results in improving patients’ survival and reducing the return of tumors.

## 2. Traditional Medical Treatments

The local treatment approach for breast cancer brain metastases (BCBM) is multimodal and it includes a combination of surgical interventions, whole-brain radiation therapy (WBRT), stereotactic radiosurgery (SRS) or fractionated stereotactic radiotherapy (fSRT). Only in some cases is it possible to perform surgery, especially for large and systematic lesions. However, postoperative radiotherapy (RT) is the better choice, since surgery alone does not guarantee a local control [14]. 

Historically, WBRT has been the standard treatment for brain metastases caused by breast cancer, but more evidence has shown that it might be less efficient for local tumors and that it is more effective for cases where there are multiple areas of disease with 5 to 10 lesions [15]. WBRT after surgical resection or radiosurgery does not significantly improve overall survival (OS). Additionally, it spreads radiation to the entire brain. However, as a palliative treatment, WBRT is highly effective and 70% of patients show symptom relief. Despite its efficacy in alleviating symptoms, its local control rate is limited [16,17]. This means that while it can significantly improve quality of life by reducing symptoms, it is less effective at controlling the growth or recurrence of brain metastases in the treated area. In their study, Chougule et al. [18] reported local control rates of 87% for patients treated with stereotactic radiosurgery (SRS) using Gamma Knife (GK), 91% for those treated with a combination of SRS and WBRT and 62% for those receiving WBRT alone. These findings indicate that local tumor control is comparatively lower for patients who undergo only WBRT. To improve local control, techniques like conformational fractionated external beam boost (SIB) have been developed, achieving control rates above 75%. Dose escalation methods, such as WBRT + SIB and WBRT followed by SRS, have shown good results [19]. Evidence demonstrated that progression outside the boost area is lower in WBRT + SIB compared to WBRT + SRS (39.4% vs. 75%), though progression in the boost area is higher in WBRT + SIB (60.6% vs. 25%) due to a higher biologically effective dose in WBRT + SRS [20]. However, WBRT can cause side effects like brain edema, hair loss and neurocognitive function (NCF) impairment [21], impacting the quality of life of patients [22]. The most common neurocognitive dysfunction is short-term memory loss [23]. NCF decline typically occurs 3–6 months after WBRT and it can be irreversible and progressive [24]. With the aim to decrease cognitive impairments, an innovative technique, called the hippocampal-avoidance technique (HA-WBRT) has been developed [25]. HA-WBRT helps preserve quality of life and studies show lower risks of neurocognitive failure, even if there are no differences in progression-free survival or overall survival compared to WBRT [26,27,28]. More details about radiotherapeutic studies will be given in Table 1.

Memantine is a neuroprotective drug and it is often administrated in patients under WBRT treatments, in order to improve cognitive functions [29]. 

Stereotactic radiosurgery (SRS) also represents an important approach in the treatment of brain tumors with metastatic lesions. It exploits intersecting beams to deliver high doses of radiation precisely to a target area, reducing the exposure to surrounding healthy tissue, especially if compared to WBRT [30]. Despite its precision, SRS can lead to late toxicity and notably radionecrosis (RN). Additionally, RN can be asymptomatic or cause symptoms like seizures, cognitive deficits, headaches and vomiting. Its incidence varies from 3% to 24%, depending on factors like radiation dose and irradiated healthy brain volume [31]. The RTOG 90-05 protocol reported RN rates of 8% at 12 months and 11% at 24 months in patients with recurrent brain metastases undergoing single-fraction radiosurgery [32]. Concurrent systemic therapies may increase RN rates, but accurate incidence data are difficult to obtain since it is difficult to distinguish between RN and tumor progression [31]. In addition, SRS is starting to be used to treat multiple brain metastases.

The effectiveness of WBRT versus SRS for patients with five or more brain metastases is still unclear. A phase III randomized trial comparing SRS to WBRT in patients with 5–15 metastases found no significant difference in median overall survival (OS) (10.4 months for SRS vs. 8.4 months for WBRT, *p* = 0.45), suggesting that avoiding WBRT might be feasible for patients with a high number of metastases [33]. Neurocognitive function also did not differ significantly between the two groups [34].

CyberKnife (CK) is a new SRT (stereotactic radiotherapy) technology and it exploits a non-coplanar and a non-isocentric circular field to treat brain cancers [14]. 

Through the integration of various angles of incidence and employing reverse planning techniques, it is possible to obtain superior target specificity while mitigating the adverse impact on surrounding healthy tissues resulting from high-dose fractionation.

To date, loco-regional treatment (LRT) with surgery remains a field of debate: results are controversial since surgery on the primary tumor could promote metastatic spread [35]. However, many retrospective studies [36,37,38] and meta-analyses [39,40] show beneficial effects of LRT in specific subsets of patients with metastatic breast cancer (MBC).

To better understand this phenomenon, Tinterri et al. [35] carried out a study to understand whether loco-regional treatment (LRT) with surgery in patients with de novo metastatic breast cancer could provide benefits and extend overall survival. Their analysis showed that there was no statistically significant survival advantage for LRT in any subgroup of patients with de novo MBC. However, a slight trend towards better recurrence outcomes was observed in triple-positive tumors. Consequently, the role of LRT in the treatment of de novo MBC remains controversial and requires further studies to identify potential beneficiaries of surgery.

These studies suggest that while surgery may theoretically exacerbate metastasis, in certain circumstances, LRT appears to confer advantages, particularly in well-defined patient subgroups. Such subsets might include individuals with specific tumor characteristics, such as hormone receptor status or molecular subtype, or those with a limited extent of metastatic disease.

**Table 1:** Radiotherapy and HER2+ breast cancer. This table shows results from studies about radiotherapy applied to HER2+ breast cancer treatments.

Study/Reference	Treatment Method	Local Control Rate (%)	Median OS (months)	Neurocognitive Function (NCF) Impact	Comments
[18]	SRS (Gamma Knife—GK)	87	7	/	High local control rate
[18]	SRS + WBRT	91	5	/	Better local control than SRS alone
[18]	WBRT	62	9	/	Lower local control rate compared to SRS and SRS + WBRT
[33,34]	WBRT vs. SRS (5–15 metastases)	/	10.4 (SRS), 8.4 (WBRT)	No significant difference between groups	Study suggests WBRT may be avoidable for patients with multiple metastases
[16,23]	WBRT + SRS; SRS alone	Limited [16], The 1-year local tumour control rate: 67% (SRS), 100% (SRS+ WBRT).	7.5 (WBRT + SRS) vs. 8 (SRS) [16].	Decline typically occurs 3–6 months after treatment and it can be irreversible and progressive [16]. Patients receiving SRS+ show a decline in learning and memory function (mean posterior probability of decline 52%) at 4 months compared to those receiving SRS alone (mean posterior probability of decline 24%) [23].	Highly effective for symptom relief
[17]	WBRT in improvement functional independence after surgery or radiosurgery for brain metastases	/	10.9 (WBRT) vs 10.7 (OBS). Note: WBRT reduced the 2-year relapse rate both at initial sites (surgery: 59% to 27%, radiosurgery: 31% to 19%, and at new sites (surgery: 42% to 23%, radiosurgery: 48% to 33%).	/	After radiosurgery or surgery for a limited number of brain metastases, WBRT reduces intracranial relapses but it does not improve functional independence or OS.
[19]	WBRT + SIB	>75	14.5	/	Novel technique to improve local control rates
[20]	WBRT + SIB; WBRT + SRS	39.4 (outside boost area), 60.6 (boost area)	24.3 (WBRT + SIB) vs. 20.3 (WBRT + SRS) Note: median intracranial PFS (WBRT + SIB): 9.1 vs. 5.9 (WBRT + SRS)	/	Lower progression outside boost area, higher progression within boost area compared to WBRT + SRS
[26]	HA-WBRT	/	6.8	Helps preserve neurocognitive function and quality of life compared to historical controls (*p* < 0.001)	Demonstrated efficacy in preserving neurocognitive function and quality of life
[27]	HA-WBRT + memantine	/	6.3	Lower risk of neurocognitive failure compared to WBRT + memantine (HR 0.74)	No differences in intracranial progression-free survival (PFS), overall
[28]	HA-WBRT without memantine	/	13.3	HA-WBRT patients without memantine show better memory preservation at 6 months, but not improved verbal fluency or executive function. Those with longer life expectancy may benefit more from this treatment.	Effective in preserving memory function survival and toxicity
[35]	FLC followed by LRT	47.5% achieved rCR	54 months	/	No significant survival advantage in any subgroup, but slight trend for better recurrence outcomes in triple-positive tumors.

FLC: Front-Line Chemotherapy, GK: Gamma Knife, HA-WBRT: Hippocampal-Avoidance Whole Brain Radiotherapy, LRT: local radiotherapy, NCF: Neurocognitive Function, OS: Overall Survival, PFS: Progression-Free Survival, rCR: Radiotherapy Complete Response, SIB: Simultaneous Integrated Boost, SRS: Stereotactic Radiosurgery, WBRT: Whole Brain Radiotherapy.

## 3. Molecular-Targeted Therapy

As previously mentioned, HER2+ breast cancer cells exhibit high expression of human epidermal growth factor receptor 2 (HER2), making them particularly susceptible to therapy that specifically targets HER2 [41]. This treatment significantly enhances the cure rate for this subtype of breast cancer.

In the context of multiple brain metastases and disease progression following local treatment, drug therapy plays a crucial role. Identifying effective therapeutic drugs is essential for managing metastatic breast cancer, particularly in cases where traditional treatment approaches may have limitations. Therefore, ongoing research is focused on developing and optimizing drug therapies to enhance patient outcomes in metastatic breast cancer cases, particularly those presenting HER2+ subtypes. Various clinical investigations have been conducted in order to study different treatments for patients who have HER2+ breast cancer and brain metastases. Concerning anti-HER2 therapy, there are mainly three categories of subdivision: large-molecule monoclonal antibodies, small-molecule tyrosine kinase inhibitor (TKI) drugs and antibody–drug conjugate (ADC) drugs [42].

### 3.1. Therapy with Monoclonal Antibodies

Trastuzumab, the first humanized monoclonal antibody targeting HER2, has revolutionized HER2+ breast cancer therapy. It blocks HER2 signaling pathways by binding to its extracellular domain, leading to cell cycle arrest and enhancing antibody-dependent cell-mediated cytotoxicity (ADCC) [43]. Preclinical research demonstrated the improved efficacy of combining cytotoxic agents with trastuzumab in HER2-overexpressing metastatic breast cancer (MBC) and adjuvant therapy, leading to rapid regulatory approvals in both settings [44,45,46,47,48,49,50,51]. Trastuzumab’s effectiveness has solidified its position as the leading therapy for HER2+ breast cancer, driving significant advancements in HER2-targeted treatments. Nonetheless, resistance and disease recurrence remain challenges for a substantial number of patients [52]. There is evidence suggesting that antibodies targeting multiple domains in HER2 exert synergistic anti-tumor effects, offering potential avenues to overcome resistance and improve treatment outcomes [53].

A second humanized anti-HER2 monoclonal antibody, pertuzumab, was developed. In contrast to trastuzumab, which targets the extracellular domain (ECD) IV of HER2, pertuzumab binds to ECD II. By doing so, pertuzumab prevents HER2 heterodimerization with HER1, HER3, and HER4, thereby blocking downstream tumor signaling pathways [54].

Studies have shown that trastuzumab is particularly effective at inhibiting cell growth in the absence of HER3 ligand [55,56]. Due to their complementary mechanisms of action and their impact on immune system-mediated anti-tumor activity through antibody-dependent cell-mediated cytotoxicity (ADCC) and/or complement-mediated cytotoxicity (CDC), combining trastuzumab and pertuzumab was hypothesized to have synergistic effects [57,58,59].

Clinical trials, such as the CLEOPATRA trial [60], investigating the combination of these two monoclonal antibodies with chemotherapy for the treatment of HER2+ breast cancer in various settings (metastatic, adjuvant, and neoadjuvant) have demonstrated superior outcomes compared to trastuzumab and chemotherapy combinations alone. These favorable results have led to the approval of pertuzumab by the FDA for use in these settings [60,61,62].

A retrospective analysis revealed that the combination of pertuzumab and trastuzumab in patients with HER2+ breast cancer brain metastases (BCBM) resulted in significantly extended overall survival, reaching 44 months compared to other groups receiving HER2-targeted therapy or non-targeted therapy (*p* < 0.001; log-rank test). Additionally, the study found that 35.7% of patients reached complete intracranial remission (CR), while 57.1% experienced partial intracranial remission (PR) [63].

Similarly, the PATRICIA study, a single-arm phase II trial, demonstrated an objective response rate (ORR) of 11% with pertuzumab in combination with high-dose trastuzumab in patients with HER2+ breast cancer and brain metastases. These results highlight the promising potential of pertuzumab-based therapy for treating this specific kind of tumor [64].

### 3.2. Therapy with Antibody-Drug Conjugated (ADC)

Trastuzumab emtansine (T-DM1) is recognized as the first drug for advanced HER2+ breast cancer therapy. Nowadays, it is the second-line treatment for this specific kind of cancer, following paclitaxel and trastuzumab [65]. T-DM1 combines trastuzumab with a cytotoxic component, DM1, a microtubule inhibitor. This compound works by inhibiting the signaling pathway downstream of HER2, promoting antibody-dependent cell-mediated cytotoxicity (ADCC), inhibiting microtubules and inducing apoptosis [66]. 

In the phase III trial, named EMILIA, patients previously treated with trastuzumab and taxane for advanced HER2+ BC were randomly assigned to receive either T-DM1 or lapatinib plus capecitabine. Notably, for patients who had central nervous system (CNS) metastases at baseline, T-DM1 treatment increased overall survival to 20.7 months, although no significant difference in median progression-free survival was observed. The CNS overall response rate was 43.6% according to RECIST 1.1 criteria; nevertheless, T-DM1 therapy is linked to an increased probability of bleeding [67,68].

In the single-arm phase IIIb KAMILLA clinical trial, stable patients with HER2+ BCBM treated with T-DM1 demonstrated an optimal objective response rate of 21.4% based on RECIST 1.1 criteria. The clinical benefit rate reached 42.9%, with median progression-free survival and overall survival standing at 5.5 months and 18.9 months, respectively. These results highlight the potential effectiveness of T-DM1 in HER2+ BCBM patients [69]. In contrast to T-DM1, trastuzumab deruxtecan (T-DXd) pairs a monoclonal antibody similar to trastuzumab with an inhibitor of topoisomerase I. Once released, these combined molecules can efficiently penetrate cell membranes, exerting a cytotoxic effect [70].

T-DXd has demonstrated robust efficacy in HER2+ BC patients who have undergone multiple lines of treatment [71].

Results from the TUXEDO-1 trial revealed encouraging outcomes among patients with HER2+ BC who have untreated BM. After therapy based on T-DXd, many patients experienced complete intracranial response (13.3%) and partial intracranial response (60%), leading to an optimal overall response rate of 73.3% according to RANO-BM criteria [72]. Patients who have undergone therapy based on T-DXd had lower probabilities of disease progression and a higher ORR. 

Furthermore, the DESTINY-Breast01 trial revealed the superiority of T-DXd over T-DM1, with a 72% reduction in disease progression or death compared to T-DM1 while maintaining a relatively tolerated side-effect profile [71]. 

In the DESTINY-Breast03 study, the T-DXd group exhibited a confirmed overall response rate of 67.4%, significantly surpassing outcomes observed with T-DM1 [73].

These results acquired even more significance in the pooled analysis of Hurvitz et al. [74]. Researchers conducted a comprehensive evaluation of T-DXd in patients with HER2+ metastatic breast cancer, particularly focusing on those with BMs.

Data from DESTINY-Breast01, DESTINY-Breast02 and DESTINY-Breast03 clinical trials were combined for this analysis. These trials collectively provided a robust dataset to assess the efficacy and safety of T-DXd in this specific patient population.

Key endpoints assessed in the study included overall survival (OS), progression-free survival (PFS), overall response rate (ORR), duration of response (DoR) and safety profile.

The status of patients with BM was classified based on the US FDA Clinical Trial Eligibility Criteria. The treated/stable BMs group included patients who had previously received CNS-directed therapy for their BMs, and their CNS disease remained stable. The untreated/active BMs group included patients who had new or progressing BMs that had not undergone a therapy directed to CNS, since their past progression.

In patients with treated/stable brain metastases, T-DXd demonstrated a higher intracranial overall response rate (ORR) (45.2%) compared to the comparator (27.6%), with a median intracranial duration of response (DoR) of 12.3 months for T-DXd. In patients with untreated/active BMs, T-DXd also showed superior intracranial ORR (45.5%) compared to the comparator (12.0%), with a median intracranial DoR of 17.5 months for T-DXd. Additionally, T-DXd exhibited numerically longer median CNS progression-free survival (PFS) in both patient groups. Overall, the safety profile of T-DXd in patients with BMs was considered acceptable, with manageable adverse events similar to those observed in the overall patient population [74].

Moreover, findings from the multicentre retrospective ROSET-BM study underscored T-DXd efficacy in HER2+ breast cancer with brain or leptomeningeal metastases. Notably, patients experienced a median progression-free survival of 16.1 months and a one-year overall survival rate of 74.9%. Intracranial response rates were particularly noteworthy in those with leptomeningeal metastases [75]. Further validation came from the DEBBRAH study, where T-DXd demonstrated an intracranial overall response rate of 46.2% in patients with active brain metastases and effectively controlled systemic lesions in 86% of cases [76].

The choice between systemic and topical therapy sequencing may influence outcomes, especially in symptomatic patients. Ongoing studies like DESTINY-12 are anticipated to provide additional insights into T-DXd efficacy for treating brain metastases in HER2+ breast cancer.

### 3.3. Therapy with Tyrosine Kinase Inhibitors

Tyrosine kinase inhibitors (TKIs) are pharmaceutical compounds designed to selectively bind to the intracellular catalytic kinase domain of HER2. By doing so, TKIs compete with adenosine triphosphate (ATP), effectively blocking its access to the kinase domain. This interference prevents the phosphorylation of tyrosine residues on HER2 and subsequent activation of downstream signaling pathways, which are vital for cancer cell proliferation and survival [77].

Lapatinib, a member of the 4-anilinoquinazoline class of TKIs, is administered orally and functions as a reversible inhibitor of both HER2 and epidermal growth factor receptor (EGFR or HER1). Its reversible binding capability allows lapatinib to competitively inhibit the kinase activity of HER2 and EGFR, thereby suppressing their signaling cascades. This inhibition is particularly advantageous in HER2-driven tumors that exhibit resistance to trastuzumab, providing an alternative therapeutic approach for these resistant cases [78].

LANDSCAPE is a single-arm phase II and multicentre trial conducted in 2013, in which the efficacy of lapatinib combined with capecitabine was demonstrated in HER2+ BC patients who had not undergone prior treatment with WBRT, capecitabine, or lapatinib. In this study, treatment was administered during cycles of 21 days. People received oral lapatinib at a dosage of 1250 mg daily, along with oral capecitabine at a dosage of 2000 mg/m^2^ daily from day 1 to day 14 of each cycle. The primary endpoint of the study was to determine the proportion of patients who achieved an objective central nervous system response. The follow-up period of the trial was more than 21.2 months and it revealed a CNS-ORR (central nervous system overall response rate) of 57.1%. Additionally, the median progression-free survival (PFS) was recorded at 5.5 months, with notably better outcomes observed in patients achieving CNS remission compared to those without remission. In conclusion, the combination of lapatinib and capecitabine demonstrated efficacy as a first-line treatment for brain metastases from HER2+ BC [79].

LANTERN, a phase II randomized trial, compared the efficacy of lapatinib plus capecitabine versus trastuzumab plus capecitabine. The results of this trial demonstrated minimal differences in central nervous system disease progression and overall progression-free survival between the two treatment regimens [80].

Neratinib, another tyrosine kinase inhibitor (TKI), has demonstrated notable activity within the central nervous system (CNS). The NALA trial investigated the efficacy of neratinib in combination with capecitabine, comparing it to lapatinib, one of the initial TKIs utilized for targeting HER2-positive CNS disease, in combination with capecitabine. Results from the study indicated improved progression-free survival in the neratinib arm, with a hazard ratio of 0.76 (95% CI, 0.63–0.93; *p* = 0.0059) [81].

Neratinib has shown effectiveness in inhibiting growth in cell lines resistant to trastuzumab, and it exhibits synergy when used in combination with trastuzumab [82,83].

In the TBCRC022, Co3 phase II clinical trial, the efficacy of neratinib plus capecitabine was investigated in patients with CNS progression following prior CNS-directed treatment. In the group not receiving lapatinib (Cohort A), the CNS overall response rate (CNS-ORR) was 49% (95% CI: 32–66%), while in the group in which lapatinib was administrated (Cohort B), it was 33% (95% CI: 10–65%). The median progression-free survival (PFS) durations for cohorts A and B were 5.5 and 3.1 months [84].

### 3.4. Tucatinib: A New Hope in Cancer Treatment

Even if PFS (Progression-Free Survival) and OS (Overall Survival) in using HER2 molecular-targeted therapies to treat breast cancer increased, addressing therapeutic resistance, especially in the context of metastatic diseases, continues to be a significant challenge in clinical practice [66,85,86]. In order to overcome resistance, the combination of monoclonal antibodies targeting HER2 with small-molecule inhibitors of HER2 was thought to be a solution. However, in some cases, this caused toxicities because of the off-target inhibition of other receptor tyrosine kinases (RTKs), such as EGFR and HER4 [87,88].

Additionally, the current antibody-based treatments approved for use have limited ability to penetrate the central nervous system (CNS), and they show reduced efficacy against CNS metastases in some cases [67,79,84]. 

It is in this context that tucatinib was developed. Tucatinib is an orally administered and selective small-molecule inhibitor of HER2. It acts by targeting the HER2 receptor with a high degree of specificity, exhibiting more than a 50-fold selectivity for HER2 over EGFR. Notably, tucatinib has the ability to penetrate the blood–brain barrier effectively [89,90,91].

In 2022, O’Brien et al. analyzed 456 molecularly characterized cell lines from various cancer types to assess the potential efficacy of tucatinib-based therapies. Unlike other HER2 inhibitors, tucatinib showed significant activity primarily in cell lines with HER2 amplification, owing to its high selectivity for HER2 over other receptors. Biomarker analysis revealed that cell lines with elevated phosphorylated HER2 and EGFR were most responsive to tucatinib. This suggests a dependence on activated HER2 signaling for optimal response. Interestingly, HER2-mutated cell lines showed low baseline levels of these markers and were unresponsive to tucatinib. These findings suggest that tucatinib may have a selective therapeutic window and could benefit patients with HER2-positive cancers characterized by HER2-driven signaling [92].

### 3.5. Therapeutic Synergy: Combination of Treatments

With the purpose of enhancing the safety of patients with HER2+ BC and the efficacy of therapy, tucatinib was explored in combination with other HER2-molecular targeted therapies in different studies. 

The HER2CLIMB clinical trial investigated the efficacy and safety of adding tucatinib to the standard treatment of trastuzumab and capecitabine in patients with HER2+ metastatic breast cancer who had previously received other HER2-targeted therapies, such as trastuzumab, pertuzumab, and trastuzumab emtansine.

Results from the trial, which included 612 patients, showed evidence of the benefits of tucatinib combination therapy. Progression-free survival (PFS) at one year was notably higher in the tucatinib combination group (33.1%) compared to the placebo-combination group (12.3%). The median duration of PFS was also longer in the tucatinib group (7.8 months vs. 5.6 months). 

Additionally, overall survival (OS) at two years was significantly improved in the tucatinib combination group (44.9%) compared to the placebo-combination group (26.6%). The median OS was extended with tucatinib (21.9 months vs. 17.4 months).

Importantly, patients with BM also experienced significant benefits from tucatinib. In this subgroup, the addition of tucatinib led to a remarkable improvement in PFS compared to placebo, with a PFS rate of 24.9% versus 0% at 1 year [91]. 

There is the possibility to combine HER2-targeted therapy with radiotherapy, Table 2 summarizes studies on HER2-target therapy associated with radiotherapy.

Liang et al. studied the impact of HER2 on breast cancer cells’ radiosensitivity. Trastuzumab increased radiation-induced cell death in high HER2+ cells, overcoming their radiation resistance [93].

In addition, trastuzumab downregulated HER2, sensitizing cells to radiation. 

Furthermore, inhibiting the PI3-K pathway enhanced trastuzumab’s radiosensitizing effects, highlighting its importance in HER2-targeted therapy and radiation response [94].

Trastuzumab can also be associated with whole-brain radiotherapy (WBRT) in HER2+ BM patients. 

In 2011, Chargari et al. conducted an analysis involving 31 HER2+ breast cancer patients with brain metastases treated with WBRT along with concurrent or continuous trastuzumab. The objective response rate (ORR) was 74.2%, the median survival time was 18 months and the median intracranial disease control time was 10.5 months [94]. 

Subsequent studies have further supported these findings, demonstrating that the combination of WBRT and anti-HER2 therapy can achieve median overall survival (OS) ranging from 12.8 to 34 months in HER2+ patients, whereas patients treated with WBRT alone have a median OS of less than or equal to 10 months [65,99,100]. Patients receiving anti-HER2 therapy along with WBRT have shown improved outcomes compared to those treated with WBRT alone. 

Moreover, combinations involving T-DM1 with radiation, high-energy focused ultrasound, macitentan or tucatinib have also yielded favorable results in the treatment of breast cancer brain metastases [65]. However, it is important to notice that T-DM1 combined with stereotactic radiosurgery (SRS) may potentially increase the risk of radionecrosis, as observed by Stumpf et al. [98].

Additionally, the blood–brain barrier (BBB) presents a challenge in delivering chemotherapeutic drugs and targeted therapies, such as trastuzumab and pertuzumab, to the central nervous system (CNS), limiting their effectiveness against brain metastases [101]. Unlike monoclonal antibodies, small-molecule tyrosine kinase inhibitors (TKIs) can penetrate the BBB and increase the concentration of the drug in CNS, potentially offering a viable therapeutic way for CNS metastases [96]. In this context, it was shown that radiotherapy can enhance BBB permeability, thus improving drug efficacy [97]. 

Unfortunately, there are still few results extremely focused on the safety and effectiveness of treatments regarding anti-HER2 therapies combined with radiotherapy. However, experts suggest that for patients with stable systemic disease and limited progression in the central nervous system that can be treated with stereotactic radiosurgery (SRS), anti-HER2 monoclonal antibodies can be continued during RT. If there is repetitive progression in the CNS over a short period, delaying SRS and changing systemic therapy may be considered as an alternative approach [102].

## 4. Innovations in HER2-Targeted Therapy

Ongoing clinical trials are investigating additional HER2-targeted therapies.

In the SOPHIA trial [103], a phase III study, margetuximab, a novel monoclonal antibody targeting HER2, was compared to trastuzumab in patients with metastatic HER2-positive breast cancer who had previously progressed on other therapies, including Kadcyla. Margetuximab’s unique Fc portion was evaluated, particularly in patients with reduced trastuzumab response due to genetic factors. Patients receiving margetuximab showed improved progression-free survival and overall response rates compared to trastuzumab, with similar adverse reaction profiles. These findings suggest margetuximab as a promising treatment option, especially for patients with suboptimal trastuzumab responses.

Trastuzumab duocarmazine is a novel ADC, and it was evaluated in the phase III TULIP trial [104] compared to a physician’s choice of treatment in patients with HER2-positive metastatic breast cancer who had received two or more prior lines of therapy or had progressed on T-DM1.

The trial demonstrated improved progression-free survival (7.0 vs. 4.9 months) and overall survival (20.4 vs. 16.3 months) with manageable adverse events. These findings suggest its potential as a promising therapeutic option for patients who have received prior treatments.

Pyrotinib is an irreversible pan-HER tyrosine kinase inhibitor and it showed superior efficacy to lapatinib when combined with capecitabine in HER2+ BC. In a phase 2 trial, pyrotinib significantly extended progression-free survival (18.1 vs. 7.0 months). Its effectiveness was consistent across patient subgroups, including those previously treated with trastuzumab, highlighting its potential as a possible option for refractory HER2+ breast cancer [105].

Challenging research is also analyzing the possibility of combining trastuzumab deruxtecan (T-DXd) with tucatinib. HER2CLIMB-04 is a phase II clinical trial designed to investigate the efficacy and safety of combining these two molecular targeted-HER2 therapies in patients with HER2+ breast cancer who have undergone previous treatments, including taxane and trastuzumab, with or without pertuzumab or have experienced disease progression within 6 months after neoadjuvant or adjuvant treatment with taxane and trastuzumab, with or without pertuzumab. Importantly, patients with brain metastases, including those with active lesions, are included in the study.

The primary objective of HER2CLIMB-04 is to evaluate the confirmed objective response rate (cORR). Secondary endpoints are key aspects, such as PFS, DOR, DCR, OS and safety assessments. The trial commenced enrollment in the USA in late 2020 [106].

Ongoing study into novel anti-HER2 antibodies, innovative ADCs, and promising combination therapies, such as PI3K inhibitors and CDK4/6 inhibitors, holds great promise for advancing treatment outcomes in HER2-positive breast cancer. These investigations aim to expand therapeutic options and enhance efficacy, potentially leading to significant advancements in patient care and outcomes.

Table 3 summarizes the ongoing research on HER2-targeted therapies and innovative studies on them.

## 5. Conclusions

The development of breast cancer brain metastases involves multiple complex pathways. However, the specific mechanisms driving this process remain poorly understood, necessitating further research efforts to better understand the specific steps.

Radiotherapy remained, in some cases, the first choice for HER2+ breast cancer and brain metastases patients’ treatment. WBRT effectively relieves symptoms, but studies demonstrated many negative effects, such as cognitive impairment. SRS offers better local control and NCF preservation, but it increases the risk of late toxicity, including radionecrosis, and it must be considered in treatment planning.

Nowadays, monoclonal antibodies, antibody–drug conjugates and small-molecule tyrosine kinase inhibitor drugs are fundamental components to slowly replace radiotherapy, since they demonstrate efficacy in controlling tumor growth and improving patient outcomes. The association of radiotherapy to HER2+ targeted therapy may also be considered in HER2+ cancer treatment.

Ongoing research into new agents and regimens offers hope for HER2+ breast cancer, emphasizing personalized and multidisciplinary approaches. Despite the need for improvement, molecularly targeted therapy demonstrates significant efficacy in treating this type of cancer.

## Figures and Tables

**Table 2:** Studies on HER2-target therapy associated with radiotherapy.

Clinical Trials on Combinational Radiotherapy	Description	Outcome	Patients	Study Status
Liang et al. [93]	Study of the impact of HER2 on BC cells’ radiosensitivity and involvement of trastuzumab	Trastuzumab increased radiation-induced cell death in high HER2+ cells and sensitized cells to radiation. Inhibiting the PI3-K pathway enhanced trastuzumab’s radiosensitizing effects	six breast cancer cell lines	Completed
Chargari et al. [94]	Trastuzumab and WBRT	ORR: 74.2%; median survival time: 18 months; median intracranial disease control: 10.5 months	31	Completed
Zhang et al. [95]	Survival benefit in BM patients after WBRT in combination with anti-HER2 therapy	The median OS longer in patients who received chemotherapy or anti-HER2 therapy after WBRT than in those who did not receive (16 vs. 6 months and 21 vs. 9 months)	60	Completed
Chien and Rugo, Fauquette et al. [96,97]	BBB challenge	TKIs can penetrate BBB [96]. RT enhances BBB permeability, improving drug effectiveness [97]	[96] Ionizing radiation were studied on an in vitro BBB model [97]	Completed Completed
Stumpf et al. [98]	Combination of T-DM1 and SRS	Patients receiving T-DM1: 39.1% developed CSRN. In contrast, only 4.5% of patients who did not receive T-DM1 experienced CSRN	45	Completed

BBB: blood–brain barrier, BC: breast cancer, CSRN: clinically significant radionecrosis, ORR: overall response rate, OS: overall survival, RT: radiotherapy, SRS: stereotactic radiosurgery, T-DM1: trastuzumab emtansine, TKIs: tyrosine kinase inhibitors, WBRT: whole brain radiotherapy.

**Table 3:** HER2+ Target Therapies. Clinical trials on therapies with monoclonal antibodies, antibody–drug conjugates, tyrosine kinase inhibitors, tucatinib and ongoing research on new agents to treat HER2+ breast cancer.

Clinical Study	Description	Outcome	Study Status	Patients
CLEOPATRA Trial [61]	Investigated pertuzumab addition to docetaxel and trastuzumab for HER2+ breast cancer patients	Median OS: 56.5 months, (pertzumab, trastuzumab and docetaxel group) Median OS: 40.8 months (trastuzumab and docetaxel group)	Ongoing	808
Retrospective Study by Bergen et al. [63]	Combination of pertuzumab and trastuzumab in HER2+ BCBM patients	TP OS: 44 months Other-HER2-targeted therapy: 17 months No-HER2-targeted therapy: 3 months	Completed	252
PATRICIA Trial [64]	Safety and efficacy of pertuzumab plus high-dose trastuzumab in HER2+ BCBM	ORR: 11%, 68% of patients experienced clinical benefit	Completed	40
**Clinical Trials on Antibody-Drug Conjugates (ADC)**				
EMILIA Trial [66]	Phase 3 trial comparing T-DM1 to lapatinib/capecitabine therapy in advanced HER2+ BC patients	Prolonged median OS (26.8 months), higher CNS ORR with T-DM1, higher probability of bleeding events Median in XL patients: 12.9 months	Completed	991
KAMILLA Trial [69]	Single-arm phase 3b trial assessing T-DM1 in stable HER2+ BCBM patients	PFS in patients with baseline BM: 5.5 months PFS in patients without BM: 7.7 months. Median OS in patients with baseline BM: 18.9 months Median OS in patients without BM: 30.0 months	Ongoing	398
TUXEDO-1 Trial [72]	Investigated T-DXd in HER2+ BC patients with untreated BM	Response Rate of 73.3%, PFS: 14 months, OS not reached	Completed	15
DESTINY-Breast01 Study [71]	Demonstrated superiority of T-DXd over T-DM1 in HER2+ BC	Median OS T-DXd patients: 29.1 months Median PFS T-DXt patients: 19.4 months Confirmed ORR in 62% of patients	Completed	184
DESTINY-Breast03 Study [73]	Showed superiority of T-DXd over T-DM1	Confirmed ORR of 67.4%. OS: at 12 months, 94.1% of T-DXt patients were alive, compared to 85.9% T-DM1 patients. PFS: at 12 months, 75.8% of T-DXd patients were alive without disease progression, compared to 34.1% on T-DM1.	Completed	524
Pooled Analysis by Hurvitz et al. [74]	Combined data from DESTINY-Breast -01, -02, -03 trials to assess the efficacy of T-DXd in HER2+ BC patients, particularly those with BMs.	CNS PFS T- DXd vs. comparator: Stable BMs: 12.3 vs. 8.7 months Active BMs: 18.5 vs. 4.0 months. Stable BMs IC-ORR T-DXd vs. comparator: 45.2% vs. 27.6% Active BMs IC-ORR T-DXd vs. comparator: 45.5% vs. 12.0%	Completed	148
ROSET-BM Study [75]	Demonstrated T-DXd efficacy in HER2+ BC with brain or leptomeningeal metastases	Median PFS of 16.1 months and one-year OS rate of 74.9%	Completed	104
DEBBRAH Study [76]	Showed IC-ORR in patients with active BM treated with T-DXd	IC-ORR of HER2+ ABC patients with asymptomatic untreated and progressing BMs was 50.0% and 44.4%, respectively. HER2+ ABC patients with stable BMs who received T-DXd had 16-week PFS rate of 87.5%.	Ongoing	21
**Clinical Trials on Tyrosine Kinase Inhibitors (TKIs)**				
LANDSCAPE Study [79]	Evaluated lapatinib/capecitabine therapy in HER2+ BC patients without prior WBRT	65.9% of patients achieved partial CNS responses. 84% of patients had a reduction in tumour volume from baseline.	Ongoing	44
LANTERN Trial [80]	Compared lapatinib/capecitabine to trastuzumab/capecitabine in HER2+ BC	CNS disease progression: 41.8% in lap-cap and 41.2% in tars-cap. PFS: 44.4% in lap-cap and 50.0% in tras-cap arms.	Completed	30
NALA Trial [81]	Investigated neratinib/capecitabine (N1C) therapy versus lapatinib/capecitabine (L1C) therapy in patients with HER2+ BC and metastases	PFC: N1C showed a 24% reduction in the risk of disease progression or death compared to L1C. OS: not statistically significant. However, N1C showed a trend toward improved OS. ORR: N1C: 32.8% L1C: 26.7%	Ongoing	621
TBCRC022, Co3 [84]	Assessed neratinib/capecitabine therapy in patients with CNS progression following prior treatment. Patients were divided in Cohort 3A (lapatinib-naïve) and Cohort 3B (lapatinib-treated).	CNS ORR 3A: 49%, CNS ORR 3B: 33%. Median PFS 3A: 5.5 months, median PFS 3B: 3.1 months. Median OS 3A: 13.3 months median OS 3B: 15.1 months.	Completed	49
**Clinical Trials on Tucatinib**				
O’Brien et al. [92]	Analyzed cell lines to assess tucatinib efficacy in HER2+ cancers	Selectivity for HER2; dependence on activated HER2 signaling	Completed	456 molecularly characterized human cancer cell lines associated with 16 different malignant histologies
HER2CLIMB Clinical Trial [91]	Investigated tucatinib with trastuzumab and capecitabine for HER2+ metastatic BM	PFS in tucatinib-combination group at 1 year: 33.1%, PFS in the placebo-combination group at 1 year: 12.3%. OS in tucatinib-combination group at 2 years: 44.9, OS in the placebo-combination group at 2 years: 26.6%.	Completed	612
**Clinical Trials on Investigational Agents**				
Margetuximab	SOPHIA Trial (Phase III) [103]	PFS in margetuximab/chemotherapy patients: 5.8 months. PFS in trastuzumab/chemotherapy patients: 4.9 months, numerical but not statistically significant OS benefit in the first group rather than in the second one (21.9 vs. 19.8 months).	Completed	536
Trastuzumab duocarmazine	TULIP Trial (Phase III) [104]	Improved PFS in patients who received trastuzumab duocarmazine rather the PC (7.0 vs. 4.9 months) and OS (20.4 vs. 16.3 months) with manageable adverse events	Completed	437
Pyrotinib	Phase II Trial [105]	Pyrotinib ORR: 78.5%, Lapatinib ORR: 57.1%. Pyrotinib median PFS: 18.1 months, Lapatinib median PFS: 7.0 months.	Completed	128

ABC: advanced breast cancer, BC: breast cancer, BCBM: breast cancer with brain metastases, BM: brain metastases, CNS-ORR: central nervous system- overall response rate, CR: complete response, IC-ORR: intracranial overall response rate, PFS: progression-free survival, ORR: overall response rate, OS: overall survival, PR: partial response, RT: radiotherapy, TKIs: tyrosine kinase inhibitors, TP: trastuzumab and pertzuzumab, T-DM1: trastuzumab emtansine, T-DXt: trastuzumab deruxtecan, WBRT: whole brain radiotherapy.
